# Supplementary material for: Modeling glioblastoma heterogeneity as a dynamic network of cell states
Source: Mol Syst Biol. 2021 Sep 16;17(9):e10105. doi: 10.15252/msb.202010105 (PMC8444284; doi:10.15252/msb.202010105)
Supplement: Supplementary file 6 — Source Data for Figure 5 [file MSB-17-e10105-s004.zip › Figure5A_sourcedata/GSEA_3071/hallmarks_stateIII.GseaPreranked.1623742540963/HALLMARK_APICAL_JUNCTION.html]

Details for gene set HALLMARK\_APICAL\_JUNCTION[GSEA]

|  || Dataset | state33071 |
| Phenotype | NoPhenotypeAvailable |
| Upregulated in class | na\_neg |
| GeneSet | HALLMARK\_APICAL\_JUNCTION |
| Enrichment Score (ES) | -0.3205677 |
| Normalized Enrichment Score (NES) | -1.6855458 |
| Nominal p-value | 0.019586507 |
| FDR q-value | 0.051976208 |
| FWER p-Value | 0.547 |
Table: GSEA Results Summary

  

Fig 1: Enrichment plot: HALLMARK\_APICAL\_JUNCTION      
 Profile of the Running ES Score & Positions of GeneSet Members on the Rank Ordered List

  

| PROBE | GENE SYMBOL | GENE\_TITLE | RANK IN GENE LIST | RANK METRIC SCORE | RUNNING ES | CORE ENRICHMENT || 1 | PFN1 |  |  | 40 | 0.481 | 0.0122 | No |
| 2 | ACTG1 |  |  | 153 | 0.371 | 0.0007 | No |
| 3 | MYL12B |  |  | 164 | 0.365 | 0.0152 | No |
| 4 | MYL9 |  |  | 293 | 0.293 | -0.0041 | No |
| 5 | YWHAH |  |  | 297 | 0.291 | 0.0087 | No |
| 6 | THY1 |  |  | 472 | -0.253 | -0.0243 | No |
| 7 | ICAM1 |  |  | 568 | -0.258 | -0.0367 | No |
| 8 | NECTIN3 |  |  | 778 | -0.268 | -0.0781 | No |
| 9 | THBS3 |  |  | 835 | -0.271 | -0.0798 | No |
| 10 | PARD6G |  |  | 840 | -0.271 | -0.0682 | No |
| 11 | MAP4K2 |  |  | 859 | -0.272 | -0.0601 | No |
| 12 | ACTN1 |  |  | 879 | -0.273 | -0.0522 | No |
| 13 | VAV2 |  |  | 963 | -0.277 | -0.0606 | No |
| 14 | KCNH2 |  |  | 1384 | -0.299 | -0.1549 | No |
| 15 | SLIT2 |  |  | 1400 | -0.300 | -0.1447 | No |
| 16 | TAOK2 |  |  | 1441 | -0.303 | -0.1409 | No |
| 17 | CALB2 |  |  | 1639 | -0.315 | -0.1770 | No |
| 18 | CDH4 |  |  | 1669 | -0.317 | -0.1696 | No |
| 19 | ADAM9 |  |  | 1870 | -0.328 | -0.2058 | No |
| 20 | ADAM23 |  |  | 1967 | -0.334 | -0.2150 | No |
| 21 | CLDN15 |  |  | 1989 | -0.335 | -0.2047 | No |
| 22 | TRO |  |  | 2029 | -0.337 | -0.1990 | No |
| 23 | SIRPA |  |  | 2072 | -0.339 | -0.1940 | No |
| 24 | NLGN2 |  |  | 2148 | -0.345 | -0.1972 | No |
| 25 | INSIG1 |  |  | 2266 | -0.354 | -0.2108 | No |
| 26 | DLG1 |  |  | 2317 | -0.359 | -0.2069 | No |
| 27 | PIK3CB |  |  | 2507 | -0.374 | -0.2381 | No |
| 28 | NECTIN1 |  |  | 2519 | -0.375 | -0.2234 | No |
| 29 | MMP2 |  |  | 2527 | -0.375 | -0.2077 | No |
| 30 | CERCAM |  |  | 2600 | -0.382 | -0.2084 | No |
| 31 | LAMB3 |  |  | 2757 | -0.395 | -0.2301 | No |
| 32 | JAM3 |  |  | 3096 | -0.430 | -0.2971 | No |
| 33 | ADAMTS5 |  |  | 3188 | -0.441 | -0.2999 | Yes |
| 34 | COL16A1 |  |  | 3197 | -0.442 | -0.2813 | Yes |
| 35 | TNFRSF11B |  |  | 3305 | -0.461 | -0.2873 | Yes |
| 36 | AMIGO2 |  |  | 3315 | -0.464 | -0.2679 | Yes |
| 37 | CADM3 |  |  | 3329 | -0.467 | -0.2494 | Yes |
| 38 | EGFR |  |  | 3433 | -0.488 | -0.2532 | Yes |
| 39 | ITGA10 |  |  | 3442 | -0.491 | -0.2323 | Yes |
| 40 | ITGA3 |  |  | 3550 | -0.526 | -0.2352 | Yes |
| 41 | SHROOM2 |  |  | 3552 | -0.527 | -0.2108 | Yes |
| 42 | ITGB1 |  |  | 3574 | -0.537 | -0.1911 | Yes |
| 43 | ITGA2 |  |  | 3589 | -0.542 | -0.1694 | Yes |
| 44 | VCL |  |  | 3603 | -0.551 | -0.1470 | Yes |
| 45 | CD274 |  |  | 3626 | -0.563 | -0.1263 | Yes |
| 46 | VCAN |  |  | 3741 | -0.660 | -0.1248 | Yes |
| 47 | LIMA1 |  |  | 3774 | -0.706 | -0.1000 | Yes |
| 48 | TGFBI |  |  | 3781 | -0.713 | -0.0682 | Yes |
| 49 | CDH11 |  |  | 3789 | -0.721 | -0.0363 | Yes |
| 50 | FBN1 |  |  | 3790 | -0.723 | -0.0025 | Yes |
| 51 | NRXN2 |  |  | 3841 | -0.814 | 0.0227 | Yes |
Table: GSEA details [plain text format]

  

Fig 2: HALLMARK\_APICAL\_JUNCTION: Random ES distribution      
 Gene set null distribution of ES for **HALLMARK\_APICAL\_JUNCTION**
